# Mindfulness Training for Improving Attention Regulation in University Students: Is It Effective? and Do Yoga and Homework Matter?

**DOI:** 10.3389/fpsyg.2020.00719

**Published:** 2020-04-29

**Authors:** Lena Wimmer, Silja Bellingrath, Lisa von Stockhausen

**Affiliations:** ^1^Department of Psychology, University of Duisburg-Essen, Essen, Germany; ^2^School of Psychology, University of Kent, Canterbury, United Kingdom

**Keywords:** mindfulness, meditation, yoga, university students, homework, attention, cognition

## Abstract

The present study examined the effects of mindfulness training on attention regulation in university students and whether the potential benefits of implementation are influenced by the yoga component of mindfulness-based interventions (MBIs) and/or by MBI homework practice. In a non-randomized trial with pre- and post-assessments, *n* = 180 university students were allocated to either mindfulness training (experimental groups), awareness activities (active control group), or no training (passive control group). Mindfulness was taught through two MBIs, one including yoga and the other excluding yoga. Attention regulation was operationalized via behavioral indicators, namely sustained attention, cognitive flexibility, cognitive inhibition, and data-driven information processing. With the exception of speed in a cognitive flexibility task, the results indicated no systematic or differential advantage arising from mindfulness training, with or without yoga, regarding the aspects of attention regulation. There was no consistent influence of homework quantity or quality. The implications for mindfulness training in academic contexts are discussed.

## Introduction

Mindfulness-based interventions (MBIs) have been demonstrated to induce benefits in physical and mental well-being (e.g., Baer, 2003; Brown and Ryan, 2003; Grossman et al., 2004; Hofmann et al., 2010; Keng et al., 2011) and cognition (e.g., Chiesa et al., 2011; Zenner et al., 2014; van Vugt, 2015). Hence, researchers and practitioners continue to apply MBIs in a variety of settings, one of which is higher education. Rationales for providing MBIs to university/college students include (1) improvement of cognitive and academic performance, (2) management of academic-related stress, and (3) personal growth (Shapiro et al., 2011).

There is growing empirical evidence concerning these rationales. Trait mindfulness has been associated with higher overall academic performance in cross-sectional studies (Scherer et al., 2017; Chiang and Sumell, 2019). Several experimental studies assigned a group of students^1^ to either 5–20-minute breathing meditation or an active or passive control condition prior to a regular university/college course and assessed retention of course content via quiz performance at the end of the session. With the exception of Baranski and Was (2019), all studies observed superior quiz performance in the mindfulness meditation group (Ramsburg and Youmans, 2014; Calma-Birling and Gurung, 2017; Bennett et al., 2018; Lin and Mai, 2018), indicating that brief mindfulness inductions can boost short-term academic performance. Some of these studies (Calma-Birling and Gurung, 2017; Lin and Mai, 2018; Baranski and Was, 2019) provided such mindfulness inductions at the beginning of each course session over several weeks and explored whether this would lead to superior long-term academic success, indexed by performance in exams that tested the content of multiple course sessions. Consistently, no benefit of brief mindfulness inductions on long-term academic success was found (see also Yamada and Victor, 2012).

This could suggest that brief mindfulness inductions, once or twice a week over the course of a term, are not intensive or extensive enough to improve long-term academic performance; not intensive enough in the sense that dosage is too low, and not extensive enough in the sense that inductions rely exclusively on breathing meditation, unlike MBIs, which typically comprise multiple components involving various formal and informal exercises, group discussions, and psychoeducational input (Baer and Krietemeyer, 2006). However, further clarification of this matter by future investigations is needed. More information is also needed on the psychological mechanisms to which the benefits of brief mindfulness inductions on short-term academic performance trace back. Given that mindfulness has been described as an awareness emerging from non-judgmental present-moment attention focus (Kabat-Zinn, 2003), processes of attention regulation appear to be pertinent here. Resonating with this, theoretical models predominantly posit attention regulation as the core of mindfulness (e.g., Bishop et al., 2004; Shapiro et al., 2006; Hölzel et al., 2011; Holas and Jankowski, 2013; Kang et al., 2013; Malinowski, 2013) such that mindfulness, first of all, means attending to the present and being capable of redirecting attention to the present if the mind wanders. In their operational definition, Bishop et al. (2004) propose that mindfulness training benefits four sub-components of attention regulation: sustained attention, cognitive inhibition, data-driven information processing, and attention switching/cognitive flexibility. The prototypical procedure of a mindfulness exercise can help to illustrate this (Bishop et al., 2004; Malinowski, 2013). At the beginning of an exercise, in particular, breathing meditation, the meditator focuses on the somatosensory sensations of the breath (i.e., the meditation object), with the intention of maintaining this focus over an extended period of time, thereby engaging **sustained attention**. This cognitive capacity is indexed by tasks whose mastery requires that one focuses one’s attention on specific stimulus properties over several minutes. The Continuous Performance Task (CPT) and the d2-R (Brickenkamp et al., 2010) are examples of such tasks (Williams and Saunders, 1997). In both tasks, respondents must monitor stimulus properties continuously so that they can respond to the target category of stimuli while avoiding errors of commission.

Thoughts, sensations, and emotions can distract the meditator from their object at any given time. Once this mind wandering is noticed, the meditator recruits **cognitive inhibition** in order to prevent further distraction. Tasks requiring one to suppress/overwrite impulsive or automatic responses, such as the Stroop (1935) or the flanker (Eriksen and Eriksen, 1974) tasks, can be used to assess cognitive inhibition. In the Stroop color-word interference task, respondents must indicate the print color of a color word. In cases where the print color is not equal to the color word, the automatic semantic processing of the word must be inhibited in order to respond accurately. In the flanker task, participants need to indicate the direction in which a centrally presented target arrow is pointing. Cognitive inhibition is required in instances where surrounding distractor arrows, which have a strong priming effect (Nieuwenhuis et al., 2006), point in the opposite direction.

Inhibition of elaborative processing helps to preserve attentional resources, which are then available for observing the current experience, a state of **data-driven** (as distinct from concept- or schema-based) **information processing**. Data-driven information processing can be operationalized through tasks that evoke a cognitive schema, while the application of this schema prevents successful administration. A visual search task in which advantageous performance is subject to spotting items in unexpected contexts (e.g., Henderson et al., 1999; see also Bishop et al., 2004) is an exemplary paradigm: if respondents trust in their cognitive schema of the depicted setting, they tend to prematurely abort their visual search and, as a result, commit an error of omission. If, instead, respondents suspend their cognitive schema of the scene and act on a data-driven basis, they will more likely perform a complete search and detect the target.

Through **attention switching/cognitive flexibility**, the focus of attention returns to the meditation object. This mental ability is assessed via tasks in which respondents have to switch their attentional scope between different stimulus properties in order to succeed. In the Wisconsin Card Sorting Task (WCST; Berg, 1948), playing cards need to be sorted by various criteria including color, shape, and number of objects shown on the card. Participants are given feedback from which they learn whether to switch to a new sorting criterion. In the number-letter task (Rogers and Monsell, 1995), number-letter combinations appear on a computer screen. When pairs are presented in the top half of the screen, participants must categorize the number as odd or even; when they are shown in the bottom half, participants are to categorize the letter as a vowel or consonant. Hence, respondents must change the focus of their attention between letters and numbers.

This set of attention regulation strategies is thought to contribute to self-regulation (Rueda et al., 2005), a construct referring to a range of abilities facilitating target-oriented behavior and appropriate reactions to mentally challenging stimuli via control of cognition, emotion, and behavior (Posner et al., 2007; Fjell et al., 2012; Zelazo and Lyons, 2012). Thus, mindfulness training is assumed to foster self-regulation via the improvement of attention regulation.

Indeed, several empirical investigations have looked at the relation of mindfulness with attention control/self-regulation. The above-mentioned study by Scherer et al. (2017) not only reported a positive correlation of trait mindfulness with academic success but also established how trait mindfulness was positively linked with time perspective, emotion regulation, and grit, all of which are processes relevant to self-regulation. Miller et al. (2019) assigned students to either 3-minute breathing meditation at the beginning of each class over one semester or to a no-intervention condition. At the end of the semester, less frequent incidents of mind wandering and lower levels of distractibility were reported by the meditation group only. Morrison et al. (2014) developed a multi-meditation MBI comprising 7 h of training over 7 weeks, which was appended to an introductory seminar for psychology undergraduates. This MBI was contrasted with a waitlist control group. Mindfulness-based benefits were found for students’ sustained attention but not for working memory. Similarly, Ching et al. (2015) investigated the effectiveness of a multi-component mindfulness meditation course that involved 18 weekly 50-minute classes, mandatory for first-year students. When compared to a passive control group, mindfulness-based benefits emerged for vigilance, whereas evidence regarding short-term memory was mixed. Helber et al. (2012) provided students participating in regular university courses with either a 10-minute meditation practice (one out of three meditation exercises was practiced in each session) plus a homework assignment over one semester or no intervention. Both groups of students showed improved executive functioning as indexed by performance in the Stroop Color and Word and Trail Making Tests, yet time spent meditating correlated with improvement in executive functioning. Taken together, initial evidence regarding the salutary effects of MBIs on students’ attentional control is promising.

Furthermore, improvement of attention regulation could contribute not only to academic success (Leland, 2015) but also to the management of academic-related stress and personal growth, which are the other two rationales for incorporating mindfulness into higher education that were mentioned by Shapiro et al. (2011, see above). High levels of stress, anxiety, and negative affect have known detrimental effects on cognitive performance and memory, with obvious implications for learning and academic performance (overview: Shapiro et al., 2011; Bennett et al., 2018). Mindful attention regulation could counteract stress-related rumination and worry in learning and testing situations (see also Perciavalle et al., 2017) by helping students gain control over distracting thoughts and redirect attention to the task at hand. This would benefit both students’ mental health and their academic success. Ergas (2015) pointed out to what extent promoting mindful attention in students has the potential to reconstruct education and foster development of the “whole person” (for a critical review of mindfulness in education see Sellman and Buttarazzi, 2019): since mindfulness is a purposeful way of attending, it is subject to an intentional choice of the individual regarding the object that they want to focus their attention on. Hence, mindfulness can (re-)establish the value of a first-person perspective in addition to the third-person perspective predominant in current educational systems. Furthermore, the present-moment focus of mindfulness can highlight the value of the “here and now,” in so far as it appreciates that learning takes place in the present moment, whereas traditional Western education tends to direct students’ attention away from the present moment in its emphasis on the need to strive for a better future. The promotion of attention regulation has also been underlined as education par excellence (James, 1890).

Although initial results suggesting such benefits from MBIs are encouraging, more rigorous investigations are needed to corroborate the evidence base and to support incorporation into higher education settings (Shapiro et al., 2011), as existing studies have so far mostly implemented one, typically passive, control group only. However, active control groups are necessary to identify specific training effects; in addition, passive control groups are valuable for detecting effects of maturation and test repetition.

Identifying the most efficient mindfulness exercises would be worthwhile for both the theory and application of mindfulness. Many extant studies relied on breathing meditation only, and when it was used as a brief induction, no improvement of long-term academic performance was reported (see above). This could indicate that breathing meditation is not the most efficient exercise for promoting attention regulation and academic success. Thus, future studies are encouraged to examine the contribution of various mindfulness exercises.

Furthermore, more evidence is needed regarding dose-response relationships. Theories predominantly consider mindfulness to be a skill that can be improved with practice (Bishop et al., 2004). The attention regulation component, in particular, is thought to benefit from regular training as it requires effortful control in the early stages of a mindfulness meditation career and becomes more and more effortless with increasing expertise (Tang and Posner, 2009; Malinowski, 2013). Thus, a positive dose-response relationship is to be expected, particularly for attention-related outcomes. The general empirical evidence, although still rather scarce (Creswell, 2017), is mixed. The following findings support the assumption of a positive dose-response relationship specific to attention-based effects: brief MBIs have been found to reduce negative affectivity and stress (Klatt et al., 2009; Schumer et al., 2018) and to promote emotional well-being (Josefsson et al., 2014) but not executive attention (Josefsson et al., 2014). Furthermore, in a randomized controlled trial comparing 30 days of mindfulness training with 30 days of brain training, Bennike et al. (2017) observed mindfulness-specific advantages for performance in the Sustained Attention to Response Task (SART); importantly, quantity of home practice was positively associated with performance gain in the mindfulness group only. Examining the empirical evidence in the field of higher education, several researchers that did not detect benefits of MBIs consequently associated null effects with lack of training intensity, i.e., insufficient practice dose (e.g., Baranski and Was, 2019), however, this awaits confirmation from targeted investigations. The results of a recent meta-analysis investigating the effects of brief mindfulness inductions on self-regulation conflict with the assumption of a positive response-dose relationship regarding effects on attention (Leyland et al., 2019). The authors concluded that short mindfulness inductions may have a direct effect on attention mechanisms but not on domains such as emotion regulation. More research would be desirable to resolve this inconsistency.

The mere dose does not seem to be the only relevant feature of mindfulness practice that explains the type and size of training effects; it also seems necessary that mindfulness meditation is undertaken with a certain level of depth or quality, as it is essential to reach a mindful state to some degree (Ireland, 2013). For instance, it is not expected that spending hours of alleged breathing meditation in an absentminded way, i.e., combining a high dose of mindfulness practice with a very low practice quality, would lead to improved attention regulation. Interestingly, the number of studies investigating meditation quality/depth is very small (Vettese et al., 2009). The existing work confirms the relevance of practice quality for achieving a healthy activity level of the autonomous nervous system (Haslam et al., 2017), reduction of psychological symptoms (Del Re et al., 2013; see also Goldberg et al., 2019) and emotional reactivity (Cahn and Polich, 2009), and improving academic outcomes (Lin and Mai, 2018). Finally, Shapiro et al. (2011) emphasize the importance of the currently underrepresented theory-driven research so that researchers can make predictions about students’ behavior and integrate individual findings into larger patterns of results.

In this article, we report a sub-project of a large trial with university students. Another sub-project of this trial that looked at mindfulness-based effects on emotion regulation and mood has been reported in Wimmer et al. (2019). Here, we focus on a distinct data set,^2^ investigating whether mindfulness training embedded into regular university seminars improves students’ attention regulation. This approach harbors the potential to further the field through the implementation of the following measures.

First, the design involved an active and a passive control group. The active control group attended activities recommended by Blackmore and Troscianko (2018) to explore the phenomenology of awareness/consciousness. Comparable to mindfulness exercises, these activities involved meta−cognition. Yet, as distinct from mindfulness training, the awareness activities entirely lacked instruction to regulate one’s attention. Hence, the active control group controlled for meta−cognitive processes unspecific to mindfulness. The passive control group attended regular classes at university only to control for the effects of test repetition, interim events, and higher education.

Second, we assessed the contribution of yoga within MBIs by comparing two MBI curricula, one including and the other excluding yoga exercises. Mindfulness-Based Stress Reduction (MBSR), the best-known MBI, consists of three key exercises, namely: breathing meditation, body scan, and yoga/mindful movement (Baer and Krietemeyer, 2006). While yoga exercises emphasize bodily movements/postures (Schmalzl et al., 2015), breathing meditation and body scan are formal meditation exercises where one aims to concentrate on a specific object, i.e., sensations of breath in breathing meditation and perceptions of specific body areas in the body scan. Because of the layout and size of classrooms, yoga exercises appear slightly more challenging in their incorporation into a university seminar as opposed to meditation. Yoga exercises often require a considerable amount of space, whereas one can remain seated at a desk during breathing meditation and body scan. Therefore, investigating whether discontinuing yoga within MBIs is linked with reduced gains in attention regulation compared with MBIs including yoga seems to be a worthwhile endeavor. Albeit both MBIs (see above) and yoga as independent techniques (Gothe and McAuley, 2015; Luu and Hall, 2016) have demonstrated benefits for attention regulation, the effects are thought to be stronger for MBIs than for yoga: mindfulness training is predominantly mental activity while, in the case of yoga, a movement component is added (Schmalzl et al., 2015). Hence, yoga requires mental resources to be partitioned into attention regulation and monitoring of body movement (and possibly further processes), so that part of these resources is not available for attention regulation. Comparable effects of both programs (MBI with vs. without yoga) would indicate that including yoga is not essential for invigorating students’ attention regulation.

Third, to investigate dose-response relationships, participants kept logs of homework practice. Since meditation depth/practice quality has been found to be positively related to academic outcomes (Lin and Mai, 2018), these logs recorded not only the duration but also the quality of training.

Fourth, a theory-driven approach was followed by deriving hypotheses on mindfulness-based effects on attention regulation from the two-component model of mindfulness put forward by Bishop et al. (2004). We expected mindfulness-related benefits on sustained attention, cognitive flexibility, cognitive inhibition, and data-driven information processing.

The following hypotheses were tested:

(1) Systematic training of attentional capacity via mindfulness training results in greater gains in cognitive performance when compared to either the metacognitive activity in the active control group or the absence of training in the passive control group. It was assumed that both control groups could show some gain in cognitive performance caused by learning effects from repeated testing and regular education at university; still, the extent of improvement should be substantially lower than that gained following mindfulness training.

Because attention regulation is more explicitly and intensively experienced through breathing meditation and body scan than by yoga, we expected that:

(2) Discontinuing yoga within an MBI course does not weaken the benefits expected for attention regulation. Thus, compared to the mindfulness training group with yoga, the mindfulness training group without yoga is expected to result in similar effects on attentional regulation outcomes.

Finally, we used moderation models to test the following research question:

(3) Is a potential change in attention regulation after mindfulness training moderated by the quantity and quality of homework practice?

## Materials and Methods

The Ethics Committee of the Department of Psychology, University of Duisburg-Essen, approved the study. All participants gave their written informed consent, and participation was rewarded with course credits. The study had a non-randomized pre-post design with two experimental groups (mindfulness training with yoga, mindfulness training without yoga), an active control group (practicing metacognition regarding awareness), and a passive control group.

### Participants

**FIGURE 1:**
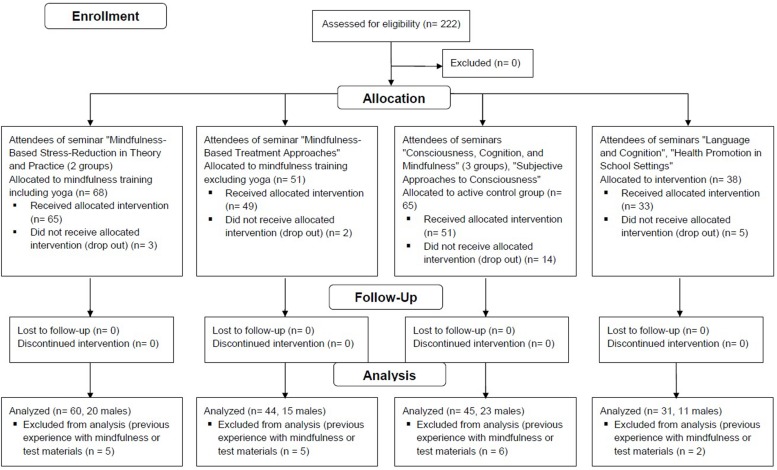
Flow of participants through the study.

Two hundred and twenty-two university students were recruited in nine psychology classes given at the University of Duisburg-Essen during two consecutive semesters. Group allocation was self-selected in so far as it depended on the classes that the students attended. Nevertheless, students were only informed about the study at the first seminar session and were free to take the class without study participation (see also Wimmer et al., 2019). The flow of participants through the study is illustrated in Figure 1. The final sample consisted of *N* = 180, with *n* = 60 participants (20 of them male) in the mindfulness group including yoga, *n* = 44 participants (15 male) in the mindfulness group excluding yoga, *n* = 45 participants (23 male) in the awareness activity group, and *n* = 31 participants in the passive control group (11 male). The mean age of the 173 participants who indicated their age was 24.92 (*SD* = 3.53).

Analyses using G^∗^Power revealed that the total sample size of *n* = 180 had a power of >0.99 to detect a global effect in a MANOVA and a power of 0.98 to detect moderation, assuming the standard 5% significance level and a medium effect size (*f*^2^ = 0.15).

### Interventions

Interventions were incorporated into psychology classes for students at the University of Duisburg-Essen, Germany. Students training to become schoolteachers were the target group of the seminars; nonetheless, students from other departments were permitted as well. Classes were provided by the authors of this article, who had engaged in mindfulness practice over a duration of three (author 1) to 9 years (author 3). Author 1 had also received formal MBSR instruction. One seminar receiving mindfulness training excluding yoga and one of the seminars for the awareness activity were taught by the first author. The second author led two classes for the mindfulness training group that included yoga and one class contributing to the passive control group. The third author held three seminars receiving the awareness activity and one seminar for the passive control group (see also Wimmer et al., 2019). Treatment fidelity was ensured by the instructors through mutual discussions to adjust the content and implementation of the seminars and interventions. Participants could not be fully blinded because interventions were embedded into seminars on mindfulness/consciousness theory. However, participants were not informed about the study aims or hypotheses before post-tests were finalized.

#### Mindfulness Training

Mindfulness training was implemented in seven bi-weekly sessions. The sessions lasted 1.5 h, during which theoretical information on mindfulness was provided (about 45 min) and mindfulness was practiced, relying on aspects of MBSR (about 45 min; see also Wimmer et al., 2019). The mindfulness training sessions drew on the well-established method of MBSR (Kabat-Zinn, 1990), however, they were strongly adapted to the setting of university classes, which is why the term MBSR is not used to name the MBI under investigation. The yoga exercises recommended by Kabat-Zinn (1990) were substituted with office yoga poses (proposed by Meyer, 2013; for the group involving yoga). In order to give room for a thorough exchange about practice experiences and feedback, the fourth session was dedicated to group discussions, with participants talking about potential challenges they had encountered. The schedule of each session can be found in Wimmer et al. (2019). Homework assignments consisted of 20 min of formal exercises (alternating between body scan and breathing meditation and – in one group – yoga) on at least 5 days of the week. Participants received taped audio instructions for body scan, breathing meditation, and (in one mindfulness group) yoga, which were taken from Meyer (2013) and Schneider (2012). In the case of body scan and breathing meditation, a short version (5 to 6 min) and a long version (15 to 17 min) were available. The short version of the body scan was newly recorded for this study, as no suitable recording was found in the literature. Instruction for the yoga poses took approximately 9 min. Since the study aimed to test the effect of practice dose, informal practices, which are hard to quantify, were not instructed. Individual conversations with the teacher were not mandatory, but participants were offered discussions on demand. Both mindfulness groups were assigned the same amount of homework, which comprised body scan and breathing meditation in the non-yoga group and body scan, breathing meditation, and yoga in the yoga group.

#### Control Groups

An active and a passive control group were implemented. The active control group practiced phenomenologically oriented awareness activities adopted from Blackmore and Troscianko (2018) to control for meta-cognitive processes non-specific to mindfulness. Here, participants were to reflect on their present state of consciousness with the help of questions including “Am I conscious now?,” “What exactly am I seeing now?,” and “Is the current state of my consciousness a mindful state?” Participants were not instructed to regulate their attention. As a homework assignment, participants were asked to engage in these awareness activities as often as possible. While the dose of the homework assignment in the active control group differed from that of the mindfulness groups, it was in line with recommendations by Blackmore and Troscianko (2018). Deviating from these in the interest of comparable doses in all treatment groups would have distorted the awareness activities to an unfeasible degree, and it would have been impossible to maintain treatment fidelity. The theoretical input in classes for these participants included concepts of self, the relationship of cognition with consciousness, various states of consciousness, and the debate on free will. The awareness activity was exclusively carried out out of class, however, participants’ experiences and potential problems were reviewed in sessions.

The passive control group did not receive any training at all in order to control for the effects of repeated assessment, intermittent events, and education at university.

### Materials

With the exception of sustained attention, which was assessed by means of a paper-and-pencil test, each of the cognitive abilities mentioned below was operationalized with one computer-based task programed using ExperimentBuilder (SR Research, 2011). Additionally, participants completed self-report questionnaires assessing the frequency and duration of their homework exercises.

#### Sustained Attention

Sustained attention was measured with the d2-R (Brickenkamp et al., 2010), a well-established paper-and-pencil test of attention and concentration: participants are presented with 14 rows, each consisting of 57 letters, d or p. Each letter has one, two, or no dashes above and/or below it, resulting in a possible range of zero to four dashes surrounding each letter (a letter has four dashes if there are two dashes above and two dashes below it). The task is to score out all ds with two dashes, while all ps as well as ds with more or less than two dashes act as distractors. The most common parameters of performance are concentration performance (number of crossed-out targets minus errors of commission), working speed (sum of crossed-out targets), and working accuracy (sum of all errors in relation to working speed). To mitigate potential test-retest effects (ClinicalTrials.gov, 2019 NCT01791348) task difficulty was increased at post-test by having participants listen to segments of radio recordings on various topics over headphones while they worked on the task. The increment of task difficulty had been planned before study commencement.

#### Cognitive Flexibility

We measured cognitive flexibility using the number-letter task (Rogers and Monsell, 1995). Stimuli consisted of number-letter pairs (e.g., “G2”) displayed in the center of the upper or lower half of a computer screen. Pairs were created by combining one out of eight numbers (2, 3, 4, 5, 6, 7, 8, and 9) with one out of eight letters (A, E, G, I, K, M, R, and U). When number-letter pairs were presented in the top half of the screen, participants were to categorize the number as odd or even; when pairs appeared in the bottom half, the letter had to be rated as vowel or consonant. The difference in participants’ reaction times (RT) was calculated for trials where the target category of a judgment from the preceding trial was repeated in the current trial (repetition trials; e.g., judge the number in preceding and in the current trial) and for trials where the target category changed (switch trials, e.g., judge the letter in the preceding trial, judge the number in the current trial). The difference in response times represents the cost of switching (Monsell, 2003): the greater the cognitive flexibility, the smaller the cost of switching.

#### Cognitive Inhibition

Cognitive inhibition was measured by employing an arrow version of the flanker task (Eriksen and Eriksen, 1974). Here, participants have to state the direction of a central target arrow as quickly as possible while disregarding six flanker arrows. The flankers are either congruent with the target (i.e., in direction of the target arrow, e.g., < < < < < < <) or incongruent (i.e., in a different direction from the target arrow, e.g., < < < > < < <). To yield a more pronounced effect of processing conflict, the flanker arrows were displayed 100 ms prior to the central target arrow (cf. Nieuwenhuis et al., 2006). More efficient cognitive inhibition is reflected in a relatively smaller discrepancy between incongruent and congruent trials: the so-called congruency effect.

#### Data-Driven Information Processing

A task based on a visual search task by the Biederman lab (Biederman et al., 1973, 1982) was used to assess the capacity for data-driven information processing (cf. Wimmer et al., 2016). Participants were to decide whether a prime object that was presented for 5 s also appeared in a subsequent image of an everyday scene (for more details on the stimuli, please refer to Wimmer et al., 2016). In 25% of all trials, the target picture exhibited the prime object in its expected position, in another 25%, it presented the object in an unexpected location, and in half of the trials, it did not include the prime object at all. Across target images, prime objects in unexpected positions were equally distributed across the four quadrants of the display. Participants received different sets of stimuli at pre- and at post-test. Superior data-driven information processing is projected to result in better ability to spot objects in unexpected positions and to accurately dismiss blank trials.^3^

#### Practice Properties

Participants of the three intervention groups (mindfulness training with yoga, mindfulness training without yoga, and awareness training) completed diaries, implemented as Word files, to yield estimates of the quantity and quality of homework. In these diaries, respondents documented how long they practiced each of the assigned exercises each day. Another section of the diaries addressed the quality of mindfulness practice using a German translation of the Toronto Mindfulness Scale (TMS; Lau et al., 2006; the translation was done by the authors and checked by a professional copy editor; it is available from the authors upon request). Since this questionnaire is a measure of state mindfulness, it did not appear to accurately assess the quality of the awareness activities. Nonetheless, participants of this group were administered the TMS to prevent imbalances between the groups that arise from reactivity to measuring instruments, e.g., effects of position and sequence. However, TMS results are reported exclusively for the mindfulness training groups. Practice quality was also assessed in open responses, which will be reported elsewhere. Each Word file diary covered a practice interval of 2 weeks, so that respondents completed five diaries in total. For more details on the assessment of practice properties, please refer to Wimmer et al. (2019).

### Procedure

The cognitive tests were conducted in a laboratory at the local university at the start and at the end of the term. Tasks were always administered in the following order: d2-R, visual search task, flanker task, and number-letter task. Testing sessions were guided by student research assistants unaware of participants’ treatment groups. Further psychometric measures on well-being and emotion regulation were collected in a paper-and-pencil format, the results of which are reported in Wimmer et al. (2019). Interventions commenced after the pre-tests had been finalized and lasted for the entire term, i.e., 12 weeks. The mindfulness classes took place every 2 weeks, while the classes on consciousness, which did not contain practice elements, were held once a week. This resulted in seven sessions for the mindfulness groups, including training units, and twelve sessions for the awareness group without training elements. The mindfulness classes took place only every 2 weeks in order to reduce students’ time burden in terms of compulsory attendance. This was done with the expectation of participants adhering to homework standards by allowing the time needed for mindfulness practice out of class. Immediately after the interventions ended, post-tests were carried out. At this point, prior experience with meditation and with test materials was recorded as well: participants responded to the item “I have engaged in regular meditation practice before the start of this seminar” by choosing “yes” or “no.” Participants selecting “yes” were considered to have an established meditation practice. Furthermore, students were asked whether they had attended previous seminars led by the first and/or the third author in which an overlapping set of assessment measures had been applied. An affirmative response indicated prior exposure to the measurement instruments of this investigation.

### Data Analysis

Only the data of participants without prior exposure to the test materials or an established meditation practice was included in the analyses. Table 1 summarizes descriptive statistics for indicators of attention regulation.

**TABLE 1:** Descriptive statistics on components of attention regulation by measures, experimental conditions, and times of testing.

	Passive control group		Awareness activity		Mindfulness training without Yoga		Mindfulness training with Yoga	
				
			(Active control group)					
	T1: *M (SD)*	T2: *M (SD)*	T1: *M (SD)*	T2: *M (SD)*	T1: *M (SD)*	T2: *M (SD)*	T1: *M (SD)*	T2: *M (SD)*
D2-R working accuracy	10.04 (9.65)	8.30 (7.70)	11.43 (8.91)	9.29 (8.43)	11.57 (10.03)	8.70 (6.58)	10.35 (7.62)	9.92 (9.65)
D2-R working speed	183.94 (35.33)	200.10.(37.27)	190.78 (35.20)	203.04 (32.21)	189.64 (28.13)	208.93 (30.46)	198.17 (33.27)	220.95 (35.15)
D2-R concentration performance	165.00 (32.94)	181.94 (28.02)	168.07 (30.35)	184.11 (34.37)	167.00 (27.00)	190.38 (28.53)	176.92 (34.48)	198.85 (37.81)
Number-Letter task reaction time	250.80 (117.25)	223.64 (111.67)	233.10 (127.16)	208.03 (102.75)	257.88 (168.62)	183.08 (80.47)	225.51 (99.30)	176.40 (98.07)
Number-Letter task response accuracy	1.99 (3.18)	1.34 (2.65)	1.23 (3.02)	1.50 (3.40)	1.67 (3.16)	1.22 (3.09)	1.43 (3.09)	1.62 (2.98)
Flanker task reaction time	93.41 (50.56)	74.71 (30.98)	73.97 (34.75)	69.56 (27.63)	93.73 (39.73)	82.22 (32.03)	89.57 (37.74)	75.68 (24.92)
Flanker task response accuracy	1.52 (2.17)	2.26 (2.83)	1.65 (1.94)	1.60 (2.71)	1.84 (2.06)	2.05 (2.20)	2.33 (8.37)	1.93 (2.96)
Visual search false alarms blank trials	0.43 (0.63)	0.40 (0.67)	0.58 (1.03)	0.51 (0.76)	0.84 (0.81)	0.49 (0.70)	0.44 (0.65)	0.49 (0.73)
Visual search correct rejections blank trials	15.23 (0.82)	15.57 (0.68)	15.16 (1.19)	15.47 (0.76)	15.02 (0.94)	15.49 (0.70)	15.19 (1.36)	15.46 (0.77)
Visual search RT blank trials	3393.85 (1568.13)	2742.76 (1292.18)	3001.74 (1555.34)	2660.98 (1370.38)	2993.16 (1474.89)	2476.14 (1241.80)	3031.14 (1584.37)	2673.32 (1346.88)
Visual search misses unexpected position	1.13 (0.86)	0.83 (0.70)	1.00 (1.02)	1.27 (1.12)	0.86 (1.04)	1.09 (0.78)	1.12 (0.93)	1.29 (0.98)
Visual search hits unexpected position	6.80 (0.85)	7.17 (0.79)	7.00 (1.02)	6.73 (1.12)	7.14 (1.04)	6.91 (0.78)	6.88 (0.93)	6.71 (0.98)
Visual search RT unexpected position	133.44 (776.00)	−58.36(632.81)	24.66 (713.21)	−115.26(594.65)	76.47 (685.16)	−87.05(683.17)	90.47 (717.57)	−2.03(683.19)

*T1 = pre-test; T2 = post-test.*

We employed the standard five percent significance level. Drop-outs were equally distributed across all groups (for exact numbers, please see Figure 1).

Performance in the d2-R (Brickenkamp et al., 2010) was analyzed with respect to working accuracy, working speed, and concentration performance, each in terms of raw scores. The data of two participants in the mindfulness training group without yoga, of one participant in the mindfulness training group with yoga, and of one participant in the active control group could not be analyzed due to comprehension problems during test administration.

Performance in the number-letter task was analyzed by contrasting switch trials with repetition trials. The data of four participants (two from the mindfulness training group with yoga, one from the active control group, and one from the passive control group) were not analyzed due to an accuracy rate below 67.1%. Response accuracy was defined as the number of correct repetition trials minus the number of correct switch trials. With regards to RT, values that were shorter than 100 ms or more than 3 SD away from the mean were deleted (2.91% of correct trials). The dependent measure was the RT of correct switch trials minus the RT of correct repetition trials.

Similar to the number-letter task, analysis of the flanker task contrasted incongruent with congruent trials. The data of two participants from the active control group could not be analyzed due to comprehension problems during test administration. Response accuracy was defined as the number of correct congruent trials minus the number of correct incongruent trials. Regarding RT, values that were shorter than 100 ms or more than 3 SD away from the mean were excluded from the analysis (1.59% of correct trials). The dependent measure was the RT of correct incongruent trials minus the RT of correct congruent trials.

The visual search paradigm was analyzed for blank trials and pictures presenting the target in an unexpected location, since these conditions demanded data-driven information processing. The data of one participant from the mindfulness training group with yoga, of two participants from the mindfulness training group without yoga, and of one participant from the passive control group could not be analyzed due to technical or comprehension problems during data collection. Response times that were more than 3 SD away from the mean were excluded (1.75% of the data). Regarding blank trials, response accuracy was analyzed in terms of correct rejections and false alarms. Regarding images presenting the target in an unexpected location, response accuracy was analyzed in terms of hits and misses. Target size and distance to the fixation cross were regressed out (for further details, please refer to Wimmer et al., 2016). The data of the present study are available in the Supplementary Material, see Supplementary Data Sheet S1.

## Results

### Effects on Cognition

Analyses of variance for each of the cognitive dependent measures and age did not detect baseline differences between groups, *p*s > 0.068. According to a χ^2^ test, the groups did not differ regarding gender, *p* = 0.230.

Hypothesis 1 (systematic training of attentional capacity via mindfulness training results in greater gains in attention regulation than the metacognitive awareness activity in the active control group or the absence of intervention in the passive control group) was tested in a MANOVA with groups (both mindfulness trainings collapsed vs. active control group vs. passive control group) as factors and residualized change scores between both time points of measurements as dependent variables. Residualized change scores were defined as standardized residuals obtained via regressing the post-test value of each dependent measure on the respective pre-test value. The MANOVA using Pillai’s trace revealed a significant effect of group, *V* = 0.25, *F*(26,304) = 1.66, *p* = 0.026. A follow-up ANOVA was significant for working speed in the d2-R, *F*(2,163) = 3.74, *p* = 0.026, however, simple contrasts with the passive controls as the reference category failed to reach significance, *p*s > 0.068. Another significant ANOVA was observed for number of hits in the visual search task, *F*(2,163) = 3.08, *p* = 0.049. According to contrasts, the passive controls improved more on hits than both the active controls, *p* = 0.035 and the collapsed mindfulness training group, *p* = 0.019. Finally, the ANOVA for RT in the number-letter task reached significance, *F*(2,163) = 5.28, *p* = 0.006. Contrasts suggested that the collapsed mindfulness group improved more strongly than the passive controls, *p* = 0.006, whereas the active controls did not differ from passive controls, *p* = 0.50. The remaining follow-up ANOVAS were insignificant: working accuracy in the d2-R: *F*(2,163) = 0.18, *p* = 0.835; concentration performance in the d2-R: *F*(2,163) = 2.70, *p* = 0.071; response accuracy in the number-letter task: *F*(2,163) = 0.14, *p* = 0.871; response accuracy in the flanker task: *F*(2,163) = 0.42, *p* = 0.656; RT in the flanker task: *F*(2,163) = 0.28, *p* = 0.757; misses in the visual search task: *F*(2,163) = 2.86, *p* = 0.060; RT for trials of the visual search task containing the target in an unexpected location: *F*(2,163) = 0.47, *p* = 0.628; correct rejections in the visual search task: *F*(2,163) = 0.44, *p* = 0.643; false alarms in the visual search task: *F*(2,163) = 0.50, *p* = 0.607; RT of blank trials in the visual search task: *F*(2,163) = 0.45, *p* = 0.640.

To test hypothesis 2 (discontinuing yoga within an MBI does not weaken the benefits for attention regulation), the two mindfulness training groups were entered as individual levels, resulting in four levels of the group factor (mindfulness training with yoga vs. mindfulness training without yoga vs. active control group vs. passive control group). Using Pillai’s trace, the MANOVA failed to detect a significant effect of group, *F*(39,456) = 1.41, *p* = 0.054.

To check whether these findings were masked by the level of seminar, which was partially confounded with the level of group (i.e., mindfulness without yoga was taught in one seminar, whereas mindfulness with yoga was spread across two seminars, awareness training was taught in four seminars, and the passive control group involved two seminars; please refer to section “Interventions”), the MANOVA was repeated with seminar (i.e., the university classes that contributed to the experimental and control groups; 9 levels) instead of the group (4/3 levels) as the factor. No significant effects were observed, *F*(104,1216) = 1.19, *p* = 0.098. Likewise, when using Pillai’s trace, the MANOVA did not demonstrate a significant effect of teacher on cognitive outcomes either (author 1 taught one seminar on mindfulness without yoga and one seminar on awareness training, author 2 taught two seminars on mindfulness with yoga and one seminar of the passive controls, and author 3 taught three seminars contributing to the active control group and one seminar of the passive controls), *F*(26,304) = 1.18, *p* = 0.250.

### Moderating Effects of Homework Quantity and Quality

Research question 3 concerned the potential moderating effects of homework quality and quantity or their interaction in bringing about mindfulness-related effects on attention regulation. Given that no reliable effects of mindfulness training on attention-related outcomes were detected, it appears especially interesting to analyze whether a certain threshold of quantity and/or quality of practice must be exceeded to bring about effects.

Descriptive analyses (for an overview of descriptive statistics, please see Table 2) were followed by two sets of moderated regressions employing the Process command in SPSS (version 3.0, Hayes, 2013). In set one (Process model 3), the dependent variable was the change score of one cognitive measure, while mean TMS sum score (W) and total duration of homework practice (Z) were entered as moderators. Group (X; mindfulness training without yoga vs. mindfulness training with yoga, with mindfulness training with yoga acting as the reference category) was the predictor. The active control group was excluded in these moderations since the TMS does not reflect homework quality in this group. Although duration and quality of homework were conceptualized as two parallel moderators, which would usually be addressed by Process model 2, Process model 3, testing a moderated moderation, was chosen; this was because the latter includes a three-way interaction of the focal predictor with both moderators on top of two-way interactions whereas Process model 2 only involves two-way interactions of the focal predictor with one moderator each but no three-way interaction. The three-way interaction of treatment group with homework duration and quality is particularly important for the data set of the experiment. It would be meaningful to see whether, for instance, a certain duration of homework is only effective if it is accompanied by a certain level of homework quality and whether this differs between intervention types. The second set of moderated regressions (Process model 1) again included the change score of one cognitive measure (Y) as the dependent variable, but there was only one moderator, namely total duration of homework practice (W). Group (X) as the predictor involved all intervention groups (active control group vs. mindfulness training without yoga vs. mindfulness training with yoga) and was coded using the indicator method, with the awareness training group serving as reference. Moderators were mean-centered in both sets of moderations.

**TABLE 2:** Descriptive statistics on homework quality (via TMS = Toronto Mindfulness Scale) and quantity by groups and times of assessment.

	Awareness activity (Active control group)	Mindfulness training without yoga	Mindfulness training with yoga
	*M (SD)*	*M (SD)*	*M (SD)*
**TMS**			
Log 1	N/A	27.44 (8.62)	26.58 (7.01)
Log 2	N/A	27.36 (7.91)	25.53 (9.06)
Log 3	N/A	29.46 (7.58)	25.42 (9.97)
Log 4	N/A	28.12 (9.35)	25.58 (11.22)
Log 5	N/A	28.95 (10.20)	25.79 (10.93)
**Average duration of homework exercise per week (Min)**			
Log 1	48.68 (120.98)	32.49 (14.35)	47.29 (38.24)
Log 2	40.43 (84.28)	95.94 (39.75)	113.07 (54.33)
Log 3	29.58 (26.97)	130.24 (50.12)	80.19 (85.68)
Log 4	31.47 (27.24)	121.19 (52.27)	116.67 (80.67)
Log 5	39.07 (60.36)	149.09 (53.31)	143.25 (68.70)

A repeated-measures ANOVA with time (Log 1, …, Log 5) as a within-subjects factor and group (mindfulness training without yoga vs. mindfulness training with yoga) as a between-subjects factor was used to test for differential developments of homework quality between the two mindfulness groups. No significant main effects or interactions on the mean sum score of the TMS (*p*s > 0.23) were observed. In regard to homework quantity, the repeated-measures ANOVA likewise included time (Log 1, …, Log 5) as the within-subjects factor and group as the between-subjects factor comprising three levels: active control vs. mindfulness training without yoga vs. mindfulness training with yoga. Main effects of group, *F*(2,146) = 37.92, *p* < 0.001, and time, *F*(2.74,400.27) = 30.72, *p* < 0.001, on the average duration of homework exercise were significant; they were also qualified by a significant interaction of group with time, *F*(5.48,400.25) = 13.66, *p* < 0.001. While the active control group showed a constant weekly practice duration, *F*s ≤ 1.80, *p*s ≥ 0.19, both mindfulness groups reported increasing weekly practice times from log 1 to log 5 except for the time between log 3 and log 4 in both groups (mindfulness with yoga: *F*s ≥ 6.38, *p*s ≤ 0.014; mindfulness without yoga: *F*s ≥ 11.68, *p*s ≤ 0.001).

For the moderation analyses, only the main effects of the moderators and interaction terms are reported since they are critical in testing the impact of homework duration and quality. Table 3 summarizes the results of the first set of moderations comparing both mindfulness interventions in terms of the impact of both homework duration and quality. The results of the second set of moderations focusing on the effect of homework duration in all three treatment groups are displayed in Table 4. The findings were, in general, mixed and of small effect size apart from the following exceptions: practice quality was strongly related to improvements in RT in the visual search paradigm when the target was in an unexpected position, *b* = −13.63. In contrast, there was an adverse relation of quality with RT in the number-letter task such that higher quality was linked with slower RT, however, it was more distinct in the mindfulness group without yoga, resulting in an interaction effect of group with TMS, *b* = 10.39.

**TABLE 3:** Moderation analyses comparing both mindfulness trainings regarding the impact of both homework duration and quality.

Dependent Measure	Moderator					
	TMS	Duration	Group × TMS	Group × Duration	TMS × Duration	Group × TMS × Duration
D2-R working accuracy	*b* = 0.12, *p* = 0.167	*b* = 0.00, *p* = 0.226	*b* = −0.10, *p* = 0.481	*b* = −0.00, *p* = 0.207	*b* = 0.00, *p* = 0.119	*b* = −0.00, *p* = 0.661
D2-R working speed	*b* = 0.97, *p* = 0.013	*b* = 0.00, *p* = 0.904	*b* = −0.35, *p* = 0.471	*b* = −0.02, *p* = 0.095	*b* = 0.00, *p* = 0.249	*b* = −0.00, *p* = 0.688
D2-R concentration performance	*b* = 0.67, *p* = 0.043	*b* = −0.00, *p* = 0.895	*b* = −0.12, *p* = 0.782	*b* = −0.01, *p* = 0.536	*b* = 0.00, *p* = 0.524	*b* = −0.00, *p* = 0.926
Number-Letter task response accuracy	*b* = 0.05, *p* = 0.574	*b* = −0.00, *p* = 0.240	*b* = −0.20, *p* = 0.094	*b* = 0.00, *p* = 0.479	*b* = −0.00, *p* = 0.473	*b* = 0.00, *p* = 0.147
Number-Letter task reaction time	*b* = −0.86, *p* = 0.688	*b* = −0.00, *p* = 0.853	*b* = 10.39, *p* = 0.014	*b* = −0.05, *p* = 0.263	*b* = 0.00, *p* = 0.947	*b* = 0.00, *p* = 0.547
Flanker task reaction time	*b* = 0.48, *p* = 0.309	*b* = 0.00, *p* = 0.827	*b* = −0.644, *p* = 0.295	*b* = 0.03, *p* = 0.007	*b* = −0.00, *p* = 0.953	*b* = 0.00, *p* = 0.388
Flanker task response accuracy	*b* = 0.01, *p* = 0.880	*b* = −0.00, *p* = 0.830	*b* = −0.10, *p* = 0.365	*b* = 0.00, *p* = 0.127	*b* = 0.00, *p* = 0.137	*b* = −0.00, *p* = 0.058
Visual search false alarms blank trials	*b* = 0.01, *p* = 0.596	*b* = 0.00, *p* = 0.960	*b* = 0.00, *p* = 0.896	*b* = 0.00, *p* = 0.640	*b* = 0.00, *p* = 0.0006	*b* = −0.00, *p* = 0.027
Visual search correct rejections blank trials	*b* = 0.00, *p* = 0.982	*b* = −0.00, *p* = 0.217	*b* = −0.02, *p* = 0.684	*b* = 0.00, *p* = 0.830	*b* = −0.00, *p* = 0.122	*b* = 0.00, *p* = 0.126
Visual search RT blank trials	*b* = −26.70, *p* = 0.133	*b* = 0.61, *p* = 0.005	*b* = 43.55, *p* = 0.117	*b* = −0.76, *p* = 0.037	*b* = −0.00, *p* = 0.906	*b* = −0.05, *p* = 0.358
Visual search misses unexpected position	*b* = 0.01, *p* = 0.630	*b* = −0.00, *p* = 0.029	*b* = −0.03, *p* = 0.323	*b* = 0.03, *p* = 0.487	*b* = −0.00, *p* = 0.048	*b* = 0.00, *p* = 0.048
Visual search hits unexpected position	*b* = −0.01, *p* = 0.630	*b* = 0.00, *p* = 0.029	*b* = 0.03, *p* = 0.323	*b* = −0.00, *p* = 0.487	*b* = 0.00, *p* = 0.048	*b* = −0.00, *p* = 0.048
Visual search RT unexpected position	*b* = −13.63, *p* = 0.036	*b* = 0.19, *p* = 0.124	*b* = 12.87, *p* = 0.153	*b* = 0.02, *p* = 0.922	*b* = 0.01, *p* = 0.575	*b* = −0.04, *p* = 0.101

**TABLE 4:** Moderation analyses comparing the effect of homework duration in all three treatment groups (mindfulness training with yoga, mindfulness training without yoga, and active control group).

Dependent Measure	Moderator		
	Duration	Group (Awareness training vs. mindfulness training with Yoga) × Duration	Group (Awareness training vs. mindfulness training without Yoga) × Duration
D2-R working accuracy	*b* = 0.01, *p* = 0.018	*b* = 0.00, *p* = 0.853	*b* = −0.00, *p* = 0.267
D2-R working speed	*b* = −0.01, *p* = 0.033	*b* = 0.01, *p* = 0.212	*b* = −0.00, *p* = 0.745
D2-R concentration performance	*b* = −0.01, *p* = 0.004	*b* = 0.01, *p* = 0.147	*b* = 0.01, *p* = 0.364
Number-Letter task response accuracy	*b* = −0.00, *p* = 0.025	*b* = −0.00, *p* = 0.806	*b* = −0.00, *p* = 0.879
Number-Letter task reaction time	*b* = −0.01, *p* = 0.320	*b* = 0.01, *p* = 0.727	*b* = 0.05, *p* = 0.079
Flanker task response accuracy	*b* = 0.00, *p* = 0.030	*b* = 0.00, *p* = 0.172	*b* = 0.00, *p* = 0.042
Flanker task reaction time	*b* = 0.01, *p* < 0.0001	*b* = −0.01, *p* = 0.081	*b* = 0.02, *p* = 0.101
Visual search false alarms blank trials	*b* = −0.00, *p* = 0.396	*b* = 0.00, *p* = 0.431	*b* = 0.00, *p* = 0.244
Visual search correct rejections blank trials	*b* = 0.00, *p* = 0.212	*b* = −0.00, *p* = 0.093	*b* = −0.00, *p* = 0.151
Visual search RT blank trials	*b* = 0.27, *p* = 0.002	*b* = 0.317, *p* = 0.194	*b* = −0.30, *p* = 0.245
Visual search misses unexpected position	*b* = −0.00, *p* = 0.538	*b* = −0.00, *p* = 0.090	*b* = −0.00, *p* = 0.406
Visual search hits unexpected position	*b* = 0.00, *p* = 0.538	*b* = 0.00, *p* = 0.090	*b* = 0.00, *p* = 0.406
Visual search RT unexpected position	*b* = 0.06, *p* = 0.147	*b* = 0.13, *p* = 0.325	*b* = 0.13, *p* = 0.346

## Discussion

The present research examined whether mindfulness training embedded into regular university seminars promotes students’ attention regulation. Initial studies had demonstrated promising results in this regard, with important implications for students’ academic success and well-being. However, the field has so far been limited by a lack of active control groups, barely existing knowledge about both the contribution of individual mindfulness exercises and dose-response effects, as well as a paucity of theory-driven research questions. Responding to these issues is vital from a theoretical perspective to uncover mindfulness-based mechanisms of action and from a practical perspective in order for MBIs to be tailored to the requirements of higher education classrooms. The current study addressed these aspects in the following ways.

Active and passive control conditions were implemented to control for the effects of meta-cognitive awareness and further unspecific effects, respectively. In addition, we assessed the incremental value of yoga in promoting attention regulation by comparing two MBI curricula, one of them including and the other excluding yoga exercises. In order to investigate dose-response relationships, participants kept logs recording not only the quantity but also the quality of homework practice. A theory-driven approach was followed by deriving hypotheses on mindfulness-based effects on attention regulation from the two-component model of mindfulness proposed by Bishop et al. (2004).

Our first hypothesis was that systematic training of attentional control, as in mindfulness training, results in greater gains in attention regulation than no systematic training, as in the active and passive control groups. This assumption was largely not confirmed. There was no indication of any systematic or differential advantage of mindfulness training, with or without yoga, regarding most aspects of attention regulation measured in the present study, with two exceptions only. A mindfulness-specific advantage was observed for a single indicator of cognitive flexibility. Furthermore, mindfulness training (and the active control group) was inferior to the passive control group regarding a single indicator of data-driven information processing. The passive controls did not receive systematic instruction in data-driven information processing, and this ability is not known to benefit from maturational processes. However, the passive control group might have been affected by a test-retest effect in the sense that they learned from the pre-test experience with the visual search task. Alternatively, considering that there was an advantage for the passive controls in a single indicator only, this could be a spurious finding.

Hypothesis two predicted that discontinuing yoga within an MBI does not weaken the benefits for attention regulation. When both MBIs were treated as separate levels, a MANOVA did not demonstrate any effect of group (passive controls vs. active controls vs. mindfulness without yoga vs. mindfulness with yoga). As no cognitive benefits that could be specifically linked with mindfulness training were found, there were no cognitive advantages that yoga could have had an impact on. Thus, hypothesis two was not strengthened by the present evidence.

In the literature, there is still insufficient evidence as to how much mindfulness practice is needed to achieve a certain benefit on students’ attention regulation. Therefore, no directional hypothesis was formulated regarding the potential moderating influence of practice quantity and quality. Instead, moderation analyses explored whether the overall duration of homework practice and/or its average quality contributed to the cognitive benefits of mindfulness training. The results did not reveal a systematic influence of invested time or quality. There were only two large-size effects of practice quality, but those were in opposite directions: a beneficial effect of practice quality on RT in one condition of the visual search paradigm in contrast to a detrimental effect on RT in the number-letter task.

The null finding regarding cognitive effects of mindfulness training is unexpected and does not match several reviews/meta-analyses from the general mindfulness literature, which consistently reported positive effects of small to medium size (e.g., Chiesa et al., 2011; Zenner et al., 2014; van Vugt, 2015). A possible reason why the present study did not bring about the hypothesized effects could lie in the context and target group of the present study, which differ from classical applications of mindfulness training. MBSR was originally developed for clinical populations, such as individuals suffering from chronic pain. Although MBSR has meanwhile been adapted for particular clinical and non-clinical target groups, it is not yet known whether its applications could be challenging in certain target groups. One variable that could be critical here is recipients’ motivation/intention to participate, as it is assumed to impact the outcomes of a mindfulness course (Shapiro, 1992; Shapiro et al., 2006). Typical participants of MBSR are intrinsically motivated to participate and engage in course activities. However, in the current study, it cannot be ruled out that many participants “volunteered” for the study to receive course credits and therefore lacked intrinsic motivation. Consequently, they might not have engaged in homework practice sufficiently. This assumption is supported by the result that practice quality in terms of TMS remained constant over the duration of the intervention period. Yet, an increase in practice quality could be expected as experience with mindfulness practice grows. Participants’ self-report of homework duration generally accorded to assignments and methodologically followed the standard procedure in MBSR courses. However, the assessment through self-report, as with any self-report measure, harbors the risk that respondents answered dishonestly, here, in the sense that they overstated the actual practice frequency, duration, and quality. So, although moderation analyses did not suggest a systematic influence of invested time or quality (see above), there remains the potential that effects were influenced by practice quantity/quality but that the self-report-based assessment method prevented the detection of such a moderation. Limitations of the assessment method could also explain the inconsistent effects of practice quality reported above. Thus, future investigations would benefit from measuring practice quantity via objective tracking methods, as enabled by mindfulness apps, for example.

A lack of intrinsic motivation and commitment to homework practices could perhaps also explain the heterogeneous pattern of findings regarding the impact of practice properties. Similarly, Ching et al. (2015; see above) considered the lack of intrinsic motivation as a potential explanation for null effects of their mandatory meditation course on stress. However, several studies (Helber et al., 2012; Morrison et al., 2014; Ching et al., 2015; Miller et al., 2019) observed benefits for students’ attention regulation, despite drawing on a population that did not appear to be particularly interested in learning about mindfulness. This could perhaps indicate that high levels of intrinsic motivation are not a necessary condition for mindfulness-based benefits for attention regulation. In this case, lack of intrinsic motivation would not be suitable to explain the current pattern of results. However, none of the existing studies explicitly monitored participant motivation, and its role should be clarified by future investigations. Studies with student populations should, therefore, assess participants’ intentions and, where possible, offer courses that do not strongly depend on extrinsic reinforcement, as this could undermine intrinsic motivation.

Alternatively, the lack of mindfulness-specific benefits for most components of attention regulation could be related to the multi-faceted nature of the MBIs implemented in the current study (Vago et al., 2019). Both curricula involved at least two formal mindfulness exercises, i.e., breathing meditation and body scan, in addition to psychoeducational input from the teacher and group discussions. Furthermore, the formal exercises combined at least two types of meditation, namely: focused attention meditation (FAM) and open monitoring meditation (OMM; terms coined by Lutz et al., 2008). In FAM, practitioners intend to sustain selective attention on a certain object, whereas OMM involves being receptive to occurrences in consciousness without focusing on a specific object (Lutz et al., 2008). The combination of these types of meditation is typical of many MBIs such as MBSR (Malinowski, 2013). FAM and OMM have been proposed to affect attention regulation in different ways, at least partially (Lutz et al., 2008; Lippelt et al., 2014). While FAM could be particularly useful for fostering sustained attention (Lutz et al., 2008), most notably among beginning practitioners, a specific benefit of OMM might include enhancement of divergent thinking (Colzato et al., 2012), so that this type of meditation could be especially useful for promoting data-driven information processing. Perhaps a visible gain in sustained attention would have required exclusive practice of FAM instead of a multi-component MBI and improvement of data-driven information processing would have necessitated more frequent implementation of OMM (see also Britton et al., 2018). In the present data set, multi-component MBIs showed an advantage for response speed in a cognitive flexibility task, potentially making the combination effective in this regard.

The present study had several advantages. The sample size was relatively large, and by employing an active and a passive control group, the design was able to control for relevant alternative processes besides the focused training of attention regulation. Furthermore, cognitive performance was measured broadly, covering diverse aspects of attention regulation that are theory-based and have been identified as sensitive to mindfulness training in previous empirical studies. Additionally, homework exercise was assessed systematically over the whole period of training and took into account quality in addition to quantity.

Study limitations resulted from trade-offs between the control facilitated by a laboratory-based study and the ecological validity of field research. Since this investigation was carried out in an actual university classroom with students taking a seminar for course credit, several concessions typical of this line of research (cf. Ramsburg and Youmans, 2014) had to be made: randomization was not possible because experimental conditions were bound to different seminars that had to be subject to students’ choice. Lacking randomization is a common issue in the field, as neither Ching et al. (2015), nor Helber et al. (2012), nor Miller et al. (2019), nor Morrison et al. (2014) were able to implement rigorous randomization procedures. Since the interventions were part of university seminars, the assignment of practice duration was to be kept constant for all participants, and individual fluctuations had to be recorded as they naturally emerged but could not be controlled by the investigators. The same limitation applies to the only other study in the field (Helber et al., 2012), which looked at the effects of homework duration.

Training dose also differed between the mindfulness groups and the active control group, both in terms of class frequency/number and homework assignment. This was due to the diverse nature of these interventions, with the result that matching of dosage would have led to an intolerably severe violation of treatment fidelity. While the dose recommended for the active control intervention is “as often as possible” (Blackmore and Troscianko, 2018), the dose usually assigned in MBIs is up to 45 min of daily formal practice on 6 days of the week (Baer and Krietemeyer, 2006). On the one hand, it is not known whether asking MBI participants to practice mindfulness “as often as possible” would result in that conventional MBI dose. On the other hand, practicing the awareness activities as often as possible usually adds up to a dose far below the typical dose of MBIs – a look at Table 2 shows that the awareness activities were on average practiced for 29 to 49 min *per week*. As a potential remedy for dosage imbalances, future investigations could use an alternative active control group that can reasonably be matched with a dosage typically used in MBIs.

Furthermore, due to the context of the study, the interventions were nested in different, partly confounded, levels, which could not be fully corrected for by additional analyses testing the influence of these levels. Finally, the fact that training sessions were given by the authors, who also designed and analyzed the study, is another limitation worth mentioning. This was inevitable due to a lack of qualified staff.

Consequently, future large-scale randomized controlled trials that purposefully manipulate the amount of mindfulness practice and take motivational factors into account are encouraged. Motivation to participate should ideally be assessed before and several times during the intervention period, so that the impact of motivational dynamics can be examined. To avoid the effects of social desirability, this could be done using an implicit measure, such as an implicit association test. To assess compliance with homework assignments, objective data, such as electronic logs from mindfulness apps, would be of advantage over self-report measures. Randomization of experimental groups could present practical difficulties in university contexts but does not seem fully impossible if the seminars belonging to the different experimental conditions can be offered at parallel time slots, and students agree to be randomly assigned to one of the seminars.

In regard to the implementation of mindfulness training in higher education, the current study shows the need for further research to precisely identify the conditions necessary to bring about positive effects on attention regulation.

## Data Availability Statement

The data set for this study can be found in the Supplementary Material accompanying this article.

## Ethics Statement

The study was reviewed and approved by the Ethics Committe of the Department of Psychology at the University of Duisburg-Essen, Essen, Germany. The participants provided their written informed consent to participate in this study.

## Author Contributions

All authors designed the study, read and approved the submitted version. LW, SB, and LS contributed to data collection. LW performed the statistical analysis and wrote the first draft of the manuscript. LS and SB commented on the draft and rewrote sections of the manuscript.

## Conflict of Interest

The authors declare that the research was conducted in the absence of any commercial or financial relationships that could be construed as a potential conflict of interest.

## Footnotes

1 “Students” within this article refers to university/college students, if not otherwise stated.

2 The only data also used by Wimmer et al. (2019) are practice variables outlined below.

3 A facial recognition task (cf. Wimmer et al., 2016) provided another indicator of data-driven information processing, with results comparable to the visual search paradigm.

## Supplementary Material

DATA SHEET S1 Data set of the present study.
